# Charitable giving: The role of framing and information

**DOI:** 10.1371/journal.pone.0288400

**Published:** 2023-07-11

**Authors:** Claudia Keser, Hartmut Kliemt, Maximilian Späth

**Affiliations:** 1 Department of Economics, University of Goettingen, Göttingen, Germany; 2 CIRANO, Montreal, Canada; 3 Department of Economics, University of Gießen, Gießen, Germany; 4 Department of Economics, University of Potsdam, Potsdam, Germany; University of Guelph, CANADA

## Abstract

We investigate how different levels of information influence the allocation decisions of donors who are entitled to freely distribute a fixed monetary endowment between themselves and a charitable organization in both giving and taking frames. Participants donate significantly higher amounts, when the decision is described as taking rather than giving. This framing effect becomes smaller if more information about the charity is provided.

## Introduction and overview

According to “Giving USA 2022: The Annual Report on Philantrophy for the Year 2021”, Americans devoted as much as $ 484,85 billion to charity in 2021 [1]. Unlike coercive redistributive measures, charitable giving is a voluntary process of two-sided matching in which the potential donor is free to give (or not) while the intended recipient is free to accept gifts (or not) [2]. However, the voluntariness of the process of charitable giving does not resolve all ethical and regulatory issues of protecting the autonomy of donors that may arise in charitable giving [3].

For instance, as numerous empirical (experimental) studies have shown, the setting of default options, which come into effect when individuals fail to make an active choice, may be of crucial importance for choice outcomes and their legitimacy [4–15]. The default setting, such as ‘donate’ as the default option, can often lead to higher donation levels. However, when individuals make an uninformed decision triggered mainly by the default setting rather than by information [16], the voluntariness of the donation may not adequately protect donor autonomy. There exists a trade-off between increasing donations through default settings (manipulation of the choice architecture) and maintaining the legitimacy of charitable giving as an informed choice.

Such a trade-off remains applicable when considering a more subtle way of influencing donation decisions: framing the choice situation. In our study, we investigate experimentally how this trade-off can be mitigated or potentially avoided in a framework where we manipulate the choice description rather than the choice architecture. Following [17] we apply a design that is based on a standard dictator game [18, 19]. As in all games of this kind, in our experiment the dictator is entitled to dispose of a fixed monetary amount. Keeping the action set of the dictator invariant, we vary, both, the information concerning the recipient and the description of the decision with which the dictator is confronted. The allocation decision is once described as passing on a monetary amount ‘intended’ for the dictator (giving frame) and once as not passing on a monetary amount ‘intended’ for the charitable organization (taking frame) [20].

In our experiment, the recipient is the *International Federation of the Red Cross and the Red Crescent* (IFRC), a charity that is relatively unknown among German students, particularly those who participated in the study. Employing a 3x2 design, we vary the level of (positive) information provided about the charity (*noinfo*; *someinfo*; *muchinfo*) and the framing (GIVE; TAKE).

Our findings show that describing the allocation decision as taking increases donation compared to characterizing it as giving; the influence of framing decreases as participants receive more information about the charity. Specifically, across all three information regimes, we observe significantly higher donations with the taking frame than with the giving frame. Furthermore, as the information level increases from *noinfo* to *someinfo* and from *noinfo* to *muchinfo*, we observe a significant increase in donations within the giving frame. Conversely, within the taking frame, we find no significant differences in donations between the information regimes.

## Modeling strategy and background

### Previous results of elementary dictator game experiments in giving/taking frames

Previous experimental studies present a mixed and inconclusive picture regarding the impact of moving from a giving to taking frame in the dictator game. While some studies involving charitable giving find no effect [17], others [21, 22] report higher donations to charities under the taking frame. Similarly, studies with student participants as recipients show conflicting results, with some observing no framing effect [23–26] and others finding higher transfers to the recipient under the taking frame than under the giving frame [27–30].

In our study, we focus solely on varying the description of the situation while keeping the dictator’s choice options and their consequences unchanged. This distinguishes our research from other studies where the action space of the dictator changes with the frame [31, 32] or where the default donation is altered but the frame remains the same [33, 34]. Previous studies related to our experiment indicate that the impact of the frame depends on the relationship between the dictator and the recipient. Specifically, higher transfers to the recipient under the taking frame are observed only when the recipient belongs to the dictator’s ingroup [35]. This suggests that group loyalties and interpersonal relations play a significant role. In the context of a WEIRD (Western, Educated, Industrialized, Rich, Democratic) society, as defined by [36, 37], where impersonal relations hold central importance, strengthening impersonal ties relies heavily on information that ‘brings’ what is socially remote (perceptionally) ‘closer’ to potential donors. Studies [38, 39] demonstrate that providing additional information about poor third-world recipients or the recipient charity increases giving by donors in WEIRD societies. This suggests that offering such information may reduce the perceived ‘moral distance’ [40] and consequently intensify the perceived ‘moral obligation to donate’, potentially resulting in higher donation levels. The notion of ‘moral obligation to donate’ alludes to a personal moral satisfaction or ‘warm glow’ derived from giving directly [41] rather than merely fulfilling an impersonal ‘moral obligation’.

In our study, we explore the interaction between increasing information about the charity to reduce ‘moral distance’ and the influence of moving from a description as giving to taking. To illustrate the connection to rational-choice-theoretical accounts of charitable giving, we incorporate experimental variables and their presumed relationships in a traditional utility-maximization framework (refer to Appendix A in S1 Appendix for detailed information).

### Charitable giving in a traditional theoretical utility-maximization model

Consider a slightly modified theoretical utility-maximization framework as originally provided in [42]. It allows for both, motivation by (impersonal) social norms of giving and a (personal) warm glow of giving [41]. The utility U of the donor who adopts the dictator role is given by

$$U=u\left(e-x\right)-\;f(x-\varphi )\;+\;\alpha g(x)$$ (1)

where, *e* is the dictator’s endowment, and *x* is the amount that the dictator donates to the charity. For *u*(*e* − *x*), which is the utility the dictator derives from material payoff to herself, it is assumed: *u*’(.) > 0, *u*”(.) < 0. The second term −*f*(.) represents the disutility the dictator suffers from deviating with her donation from the contextually prevailing giving norm, *φ*; assuming that −*f*(.) ≤ 0 is strictly concave in *x* and adopting its maximum (i.e., its minimum in absolute terms) when the dictator fully complies with the norm (x = *φ*). The assumption here is that norm compliance is associated with positive feelings and violation of norms with negative feelings. The third term relates to the ‘warm glow’ of giving [41, 43], with *g*’(.) > 0 and *g*”(.) < 0 (for simplicity assuming that the warm glow of not taking is identical to the warm glow of giving). The parameter, *α* ≥ 0, represents the intensity of the warm glow of giving. Following [38], the parameter *α* can be increased by moral-distance-reducing information about the charity.

In a standard dyadic dictator game, the typical assumption is a norm of giving 50 percent of the endowment if the recipient has no own endowment [44]. If the recipient is a charity, the norm might be higher. We follow an observation of [28] who find that an allocation that leaves the recipient (in their case another person) with less than half of the endowment is perceived as less socially appropriate under the taking frame than under the giving frame. Assuming that, below 100 percent, whatever the norm of giving in a giving framework, the norm is higher in a taking framework, it becomes straightforward to demonstrate theoretically (see Appendix A in S1 Appendix) that a utility-maximizing dictator’s donation changes in direct relationship to–though by less than–a change in the giving norm. More specifically the theoretical model yields the hypotheses: (H1) ceteris paribus, higher donations under the taking than under the giving frame; (H2) ceteris paribus, higher donations, the more information is provided; and–our main point–(H3) a smaller (taking versus giving) frame effect, the more information is provided.

In terms of practical advice to ‘moral’ charities, which–other things being equal–prefer both higher donations and better-informed donors, (H2) suggests combining a giving frame with extensive information efforts. However, (H1) indicates that–other things being equal—switching to a taking frame could increase donations. According to (H3) the relative advantage of the taking over the giving frame will decline with increased information, and increases in legitimacy may then outweigh the (remaining) opportunity costs of donations forgone.

## Experimental design

We conduct a dictator game experiment [18, 19] with a charity as the recipient [20]. Donations in our experiment go to an organization that is rather unknown among students in Germany: the *International Federation of the Red Cross and Red Crescent* (IFRC). Thus, our experiment differs from [17, 22] in two respects. First, in these studies, participants could choose the recipient of the donation from a list of well-known charities. Second, even though the *German Red Cross* is part of the IFRC and the two share common goals, our participants have significantly less knowledge about the IFRC than about the well-known *German Red Cross*. We elicited the self-reported knowledge of both the IFRC and the *German Red Cross*, each based on a Likert scale from 1 (very little knowledge) till 7 (very much knowledge). We found significantly different averages of 1.91 for the IFRC and 3.59 for the German Red Cross (Wilcoxon signed-rank test: N = 239, z = 12.153, p = 0.000). Using the largely unknown IFRC as the recipient in our experiment, provides room to vary the information about the worthiness of the organization.

Our treatment variation follows a 3x2 between-subjects design. Along the first dimension, we vary the participants’ information regarding the charity as potential recipient of their donations (that might be related to the *α* of the warm-glow component in our utility model). Along the second dimension, we vary the frame between GIVE and TAKE (and thus the giving norm *φ* in our utility model). While the participants are still sitting in the waiting room, we instruct them about some general lab rules together with the information that, contingent on the outcome of the experiment, money might be transferred to a charity after the experiment. Depending on the treatment, we provide more or less information on the charity. In the treatments GIVE-*noinfo* and TAKE-*noinfo*, we inform participants merely about the name of the charity and state that the *German Red Cross* is part of this organization. In the treatments GIVE-*someinfo* and TAKE-*someinfo*, we provide some additional information to increase the positive image of the charity. This information is taken from the official website of the IFRC and includes the size of the organization, the URL of its website, its key areas of work and functions. An experimenter reads the information aloud to the participants. In the treatments GIVE-*muchinfo* and TAKE-*muchinfo*, we provide the name of the charity, read the information and, additionally, show a video to the participants (Title: “Rotkreuz-Grundsätze”. URL: https://www.youtube.com/watch?v=rVfOdY30miI. Uploaded by “Markus Hechenberger” on Jan 20^th^, 2014. Duration of 3:20 minutes. 8.075 views on April 8^th^, 2020). The video was produced by the *Austrian Red Cross*. It presents the seven fundamental principles of the IFRC, both in writing and read aloud in German language. The video includes some background music and seven pictures that display typical activities of the IFRC. We acknowledge that presenting the video might have emotional effects additional to its informational impact. These emotional effects might further strengthen the warm-glow of donating. The presentation of the read out additional information as well as that of the video significantly increased the participants’ self-stated knowledge of the IFRC. The self-reported knowledge of the IFRC increased from the *noinfo* over the *someinfo* to the *muchinfo* environment. The difference between the *noinfo* and *muchinfo* environment is statistically significant (Wilcoxon rank-sum test: N = 161, z = 3.019, p = 0.003). A transcript of all instructions and charity information provided to the participants in the waiting room can be found in Appendix B in S1 Appendix.

From the waiting room, we guide the participants to their randomly assigned private cubicles, where they find their endowments of ten euros (Appendix B in S1 Appendix additionally provides photos of a private cubicle and a typical presentation of the money). On a computer screen we present further instructions to the participants. One half of the participants in a session is privately informed that the money is dedicated to them unless they decide otherwise (giving frame: GIVE-*noinfo*, GIVE-*someinfo*, GIVE-*muchinfo*). They can freely decide to decrease their initial amount and thereby increase the amount going to the charity. Typing in ‘0’ leads to a zero donation. The other half of the participants is informed that the money is dedicated to the charity unless they decide otherwise (taking frame: TAKE-*noinfo*, TAKE-*someinfo*, TAKE-*muchinfo*). The participants can freely choose to decrease the initial amount of the charity’s money in order to increase their own. Typing in ‘0’ leads to a donation of the full amount.

The donation decision is embedded into a questionnaire to be answered during the session. Irrespective of their decision to donate or not, participants have to wait thirty seconds until they can exit the decision stage. This strongly reduces differences in transaction costs between treatments. Participants are aware of the fact that the opportunity to donate arises only once. The questionnaire is longer than a usual post-experimental survey in order to extend the experiment to a duration of about 45 minutes. Critical inquiries, which might potentially prime prosocial behavior, are placed after the decision. We do not use words such as ‘taking’, ‘giving’ or ‘donation’ (resp. their German language equivalents), neither before nor during the decision process.

The donation process is double blind in the sense that neither the charity nor other participants can observe the amount contributed by any individual participant. The experimenters are unable to relate donations to names or faces of the participants. Curtains make sure that the participants’ decision making is unobserved. Payment is conducted by the participants themselves. Participants find the endowment split into three 2-euro, two 1-euro, five 0.20-euro and ten 0.10-euro coins; that is, they may make any give or take decision between zero and 10 euros in increments of 0.10 euro. To muffle sounds, the money is placed upon a matting. After the experiment, participants take the money that they assigned to themselves. Donated money is left on the table. Participants receive no additional show-up fee. They fill in a receipt for the amount that they assigned to themselves, fold it and put it into a box. The instructions make it clear that only persons unfamiliar with the purpose and the design of the experiment will handle the receipts for accounting purposes.

We conducted our laboratory experiment, programmed in zTree [45], in 2017 till 2019 at the University of Göttingen. We invited randomly selected students from our subject pool via ORSEE [46]. No specific IRB-approval was necessary for running this standardized economic experiment at the Göttingen Laboratory of Experimental Economics. All experiments that are run at the Laboratory for Experiments in Economics (LEINE) at the University of Göttingen comply with the policies in place for the use of the laboratory, which have been developed in accordance with the standards of the field and ensure the protection of the rights and welfare of the participants. Consent to voluntarily participate in economic experiments is expressed by registering in our ORSEE database. Subjects are requested to read an online consent form and agree (by clicking a button) with its terms. They are informed that they may take part in a study on decision making, that participation is voluntary and that they may leave the experiment at any time. They are also guaranteed the anonymity of the data generated in the experiment.

For this specific experiment subjects received no separate show-up fee. Instead, they could freely take, from the 10 euros in front of them, any feasible amount for themselves. The instructions said that, for their participation, they can earn money depending on their own decisions.

In total, 239 participants took part in 22 sessions. On average there were 40 participants in each treatment (with a range from 35 to 45). Within each session, the treatments varied along the frame dimension. The variation regarding the information dimension took place between sessions. The average share of females was 54 percent. The average age of participants was 24 years of age. No significant differences between information treatments with respect to these characteristics can be detected (gender: Fisher’s exact test, p = 0.208; age: Kruskal-Wallis test, chi2 = 1.955, p = 0.855). In thirteen cases, the indicated donation did not coincide with the amount of money left in the cubicle. If the participant mentioned having made a mistake when reporting her or his decision, we base the analysis on the actual donation (amount of money left in the cubicle). Otherwise, we continue working with the declared donation decision.

## Experimental results

We denote the Wilcoxon rank-sum test as *rank-sum test* and the Fisher’s exact test as *exact test*. All tests are two-sided and we require p = 0.05 for significance.

**Fig 1:**
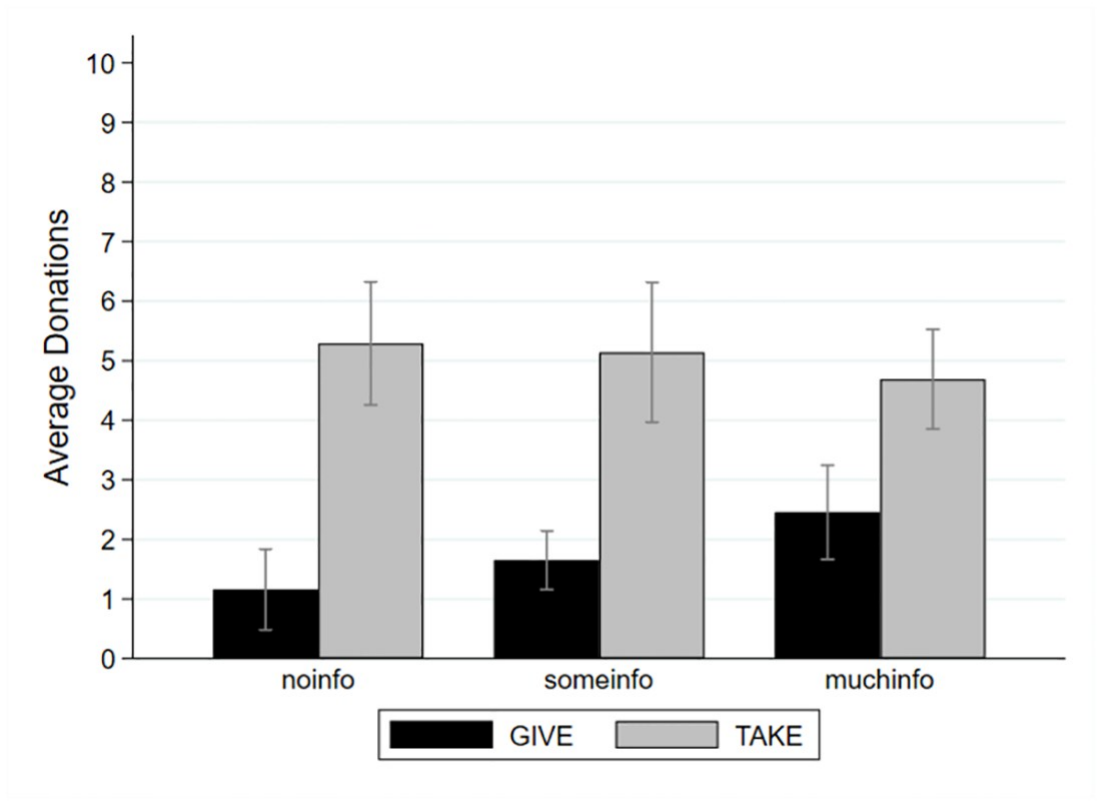
Average donations per treatment. In euros, by frame (GIVE / TAKE) and information (*no*, *some*, *much*), with 95% confidence intervals.

As illustrated in Fig 1, the average donations to the IFRC vary substantially between treatments: In the *noinfo* environment, participants on average donate 11.6 percent of their endowment of ten euros in GIVE, while they donate 52.9 percent in TAKE. In the *someinfo* context, they donate 16.5 percent of the endowment in GIVE and 51.4 percent in TAKE. In the *muchinfo* situation, they donate 24.6 percent of the endowment in GIVE, while they donate 46.9 percent in TAKE. Note that the average donations in GIVE-*noinfo* and GIVE-*someinfo* are below the average transfer of 28 percent of the endowment observed in a meta-study [47], while they are similar to this reference point in GIVE-*muchinfo*. This suggests that in our experiment, where the recipient is a relatively unknown charity, the provision of information plays a crucial role since it brings our results closer to those in the literature, where the recipient is less “distant” (i.e., another student participant or a well-known charity). Still, we acknowledge that we cannot rule out the possibility of a negative spillover effect in the sense that participants, who donated much in the experiment, donate less at later points in time outside of the lab.

**Fig 2:**
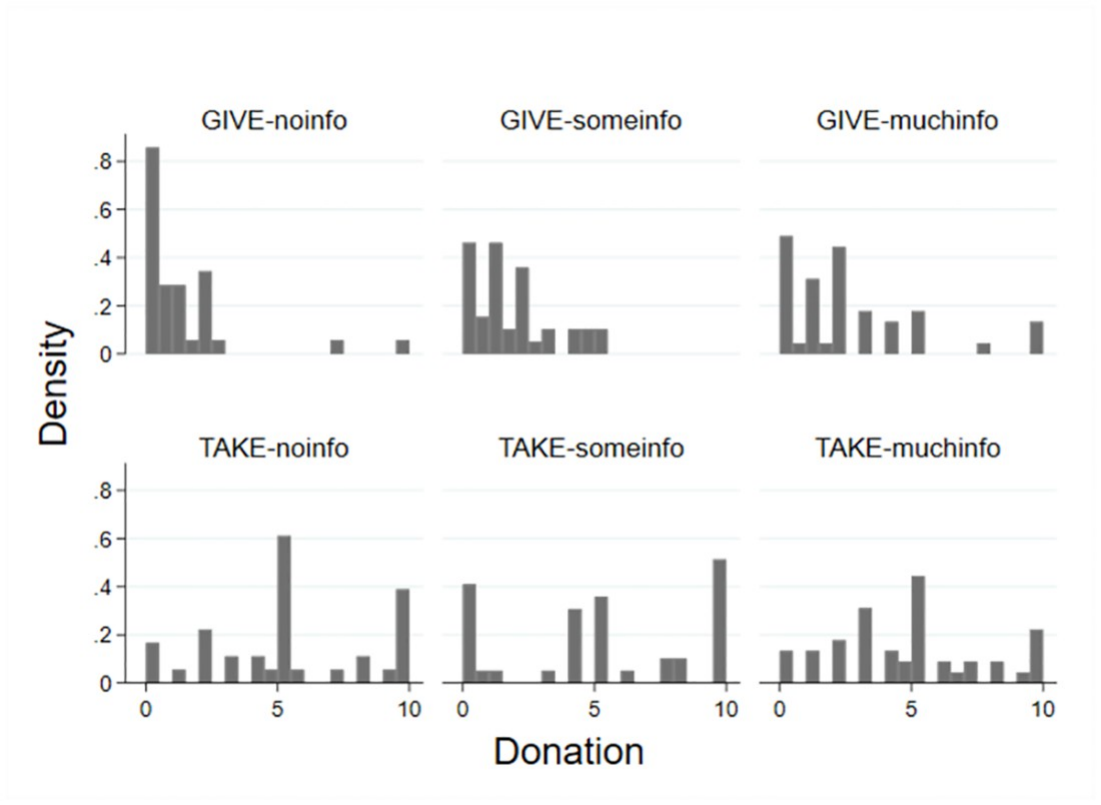
Histogram of donations. In euros, by frame (GIVE / TAKE) and information (*no*, *some*, *much*), bin width = 0.5.

In support of H1, we observe that, irrespective of the information environment, donations are on average significantly higher under the taking than the giving frame (rank-sum tests: GIVE-*noinfo* vs TAKE-*noinfo z = 5*.*453* p = 0.000; GIVE-*someinfo* vs TAKE-*someinfo* z = 3.854 p = 0.000; GIVE-*muchinfo* vs TAKE-*muchinfo z = 4*.*028* p = 0.000). The histogram of donations in Fig 2 illustrates the systematically larger donations under the taking than under the giving frame.

With respect to H2, we find no statistical evidence for an effect of the variation in information on average donations under the taking frame (Kruskal-Wallis test: chi2 = 0.755 p = 0.685). This result is confirmed by pairwise treatment comparisons (rank-sum tests: TAKE-*noinfo* vs TAKE-*someinfo z = 0*.*268* p = 0.788; TAKE-*noinfo* vs TAKE-*muchinfo* z = 0.520 p = 0.603; TAKE-*someinfo* vs TAKE-*muchinfo z = 0*.*878* p = 0.380). Under the giving frame, however, we do find statistical evidence of information effects (Kruskal-Wallis test: chi2 = 9.040 p = 0.011). Specifically, donations significantly increase when (in comparison to the *no* information domain) *some* information or *much* information is provided (rank-sum tests: GIVE*-noinfo* vs GIVE-*someinfo z = 2*.*141* p = 0.032; GIVE-*noinfo* vs GIVE-*muchinfo z = 1*.*083* p = 0.005). Only the difference in donations between *much* information and *some* information is statistically insignificant (rank-sum test: GIVE-*someinfo* vs GIVE- *muchinfo z = 2*.*841* p = 0.279). Altogether, we find support for Hypothesis 2 on the positive impact of information on donations for the giving frame but not for the taking frame.

With respect to H3, we do observe that the impact of the taking frame decreases with the information provided. Relative to the giving frame, the taking frame increases donations by 41 percentage points (of the endowment) in the *no* information environment, by 35 percentage points in the *some* information domain, and by only 22 percentage points in the *much* information environment. Table 1 presents the results of two ordinary least-squares regressions on donation. In a model without interaction terms, exhibited in column (1), we find a positive coefficient of the taking frame dummy (p < 0.001, baseline is the giving frame). The dummies for both types of additional information (pooled over both frames) are not statistically different from zero (baseline is no information). The regression results presented in column (2) exhibit that the coefficient of the interaction term between *some* information and the taking frame is not significantly different from zero. Importantly, we find the interaction between *much* information and the taking frame to have a significantly negative coefficient (p = 0.032). In support of Hypothesis 3, this suggests that providing *much* information decreases the frame’s impact on donations.

**Table 1:** Ordinary least-squares linear regression on donation.

Donation	(1)	(2)
Taking frame	3.207*** (0.359)	4.132*** (0.657)
*Some* information	0.164 (0.456)	0.492 (0.644)
*Much* information	0.342 (0.442)	1.298* (0.623)
*Some* information x taking frame		-0.642 (0.907)
*Much* information x taking frame		-1.898* (0.878)
Constant	1.626*** (0.377)	1.157* (0.467)
N	239	239
R^2^	0.254	0.269
F	F(3, 235) = 26.64	F(5, 233) = 17.18

Note: Standard errors in parentheses. Reference category for the taking frame: giving frame. Reference category for *some* information jointly with *much* information: *no* information.

* p < 0.05,

** p < 0.01,

*** p < 0.001.

## Discussion

The time honored maxim of ‘volenti not fit iniuria’ or that those who have voluntarily engaged a commitment cannot be wronged by what they have ratified by their own acts of will is a central ethical principle underlying the ‘private law society’ [48]. The maxim is almost routinely invoked by those who insist that voluntary dealings, private acts of exchange for a ‘quid pro quo’ as well as acts of charitable giving should be respected by politics. But there can be a legitimate role for regulatory policies if the architecture or framing of the choice situation or the information available in it can be manipulated.

Such regulations are widely deemed neccessary when service providers on the internet ask their clients for permission to use data that are non essential for rendering the service. In such commercial cases regulators prevent providers from exploiting default biases by setting default options to the potential disadvantage of their clients. Yet, when charities ask for donations it is less widely accepted that the regulator should prevent reliance on decision biases to increase donations.

The justification for such different regulatory demands need not be based on the charitable purpose it may also refer to the distinction between interventions into the architecture and the framing of the choice situation. Framing effects emerge from expressing identical facts ‘in other words’ but do not involve the choice architecture itself. Our experiment investigates a pure framing effect. According to the cover story as expressed by the experimental instructions, we have two cases: in case 1 the money on the mat is ‘intended’ for the charity, and in case 2 the money on the mat is ‘intended’ for the participant. In both cases, the participant must type in a figure between 0€ and 10€ to be transferred of what, in case 1, is ‘intended’ for the charity to the participant (taking frame) or of what, in case 2, is ‘intended’ for the participant to the charity (giving frame).

Our experimental results demonstrate that without a more problematic change of the choice architecture, framing otherwise identical choice alternatives as ‘taking’ rather than as ‘giving’ may lead to higher donations. We also find that additional favorable information reduces the framing effect of take-versus-give. In other words, we observe that the trade-off between increasing donations by the (‘manipulative’) framing of the choice situation and the legitimation derived from the voluntariness of the act of giving as an informed choice may not be fully avoided but largely mitigated by better information. That is, relying on framing can avoid the more significant objections against exploiting a human choice making bias by intervention in the choice architecture and still ‘nudge’ choice makers in a desired direction [49].

## Supporting information

S1 Appendix (DOCX)

S1 Data (XLSX)

S2 Data (DTA)

## References

[1] The Giving Institute. Giving USA: $484.85 billion—In 2021, Americans gave $484.85 billion to charity, a 4.0% increase over 2020. Adjusted for inflation, total giving remained relatively flat, with -0.7% growth. 2022 June [Cited 2022 October 11]. https://givingusa.org/wp-content/uploads/2022/06/GivingUSA2022_Infographic.pdf

[2] Roth AE. Who Gets What—and Why: The Economics of Matchmaking and I Design. Eamon Dolan/Houghton Mifflin Harcourt; 2015.

[3] Rodríguez-Arias D, Molina-Pérez A, Hannikainen IR, Delgado J, Söchtig B, Wöhlke S, et al. Governance quality indicators for organ procurement policies. PLOS ONE 2021; 16(6):e0252686. doi: 10.1371/journal.pone.0252686 34086783 PMC8177644

[4] Samuelson W, Zeckhauser R. Status quo bias in decision making. J Risk Uncertainty 1988; 1(1):7–59.

[5] Park CW, Jun SY, Macinnis DJ. Choosing What I Want versus Rejecting What I Do Not Want: An Application of Decision Framing to Product Option Choice Decisions. Journal of Marketing Research 2000; 37(2):187–202.

[6] Madrian BC, Shea DF. The Power of Suggestion: Inertia in 401(k) Participation and Savings Behavior. The Quarterly Journal of Economics 2001; 116(4):1149–87.

[7] Johnson EJ, Bellman S, Lohse GL. Defaults, Framing and Privacy: Why Opting In-Opting Out Marketing Letters 2002; 13(1):5–15.

[8] Johnson EJ, Goldstein D. Medicine. Do defaults save lives? Science 2003; 302(5649):1338–9. doi: 10.1126/science.1091721 14631022

[9] Choi J, Laibson D, Madrian B, Metrick A. For Better or For Worse: Default Effects and 401(k) Savings Behavior. Cambridge, MA; 2001.

[10] Abadie A, Gay S. The impact of presumed consent legislation on cadaveric organ donation: a cross-country study. J Health Econ 2006; 25(4):599–620. doi: 10.1016/j.jhealeco.2006.01.003 16490267

[11] Beshears J, Choi JJ, Laibson D, Madrian BC. Chapter 3 The Importance of Default Options for Retirement Saving Outcomes: Evidence from the USA. In: Kay SJ, Sinha T, editors. Lessons from Pension Reform in the Americas. Oxford University Press Oxford; 2007. p. 59–87.

[12] Carroll GD, Choi JJ, Laibson D, Madrian BC, Metrick A. Optimal Defaults and Active Decisions. The Quarterly Journal of Economics 2009; 124(4):1639–74. doi: 10.1162/qjec.2009.124.4.1639 20041043 PMC2798815

[13] Chapman GB, Li M, Colby H, Yoon H. Opting in vs opting out of influenza vaccination. JAMA 2010; 304(1):43–4. doi: 10.1001/jama.2010.892 20606147

[14] Chetty R, Friedman JN, Leth-Petersen S, Nielsen TH, Olsen T. Active vs. Passive Decisions and Crowd-Out in Retirement Savings Accounts: Evidence from Denmark *. The Quarterly Journal of Economics 2014; 129(3):1141–219.

[15] Blumenstock J, Callen M, Ghani T. Why Do Defaults Affect Behavior? Experimental Evidence from Afghanistan. American Economic Review 2018; 108(10):2868–901.

[16] Everett JA, Caviola L, Kahane G, Savulescu J, Faber NS. Doing good by doing nothing?: The role of social norms in explaining default effects in altruistic contexts. Eur. J. Soc. Psychol. 2015; 45(2):230–41.

[17] Grossman PJ, Eckel CC. Giving versus taking for a cause. Economics Letters 2015; 132:28–30.

[18] Forsythe R, Horowitz JL, Savin NE, Sefton M. Fairness in Simple Bargaining Experiments. Games and Economic Behavior 1994; 6(3):347–69.

[19] Kahneman D, Knetsch JL, Thaler RH. Fairness and the Assumptions of Economics. J BUS 1986; 59(S4):285.

[20] Eckel CC, Grossman PJ. Altruism in Anonymous Dictator Games. Games and Economic Behavior 1996; 16(2):181–91.

[21] Zarghamee HS, Messer KD, Fooks JR, Schulze WD, Wu S, Yan J. Nudging charitable giving: Three field experiments. Journal of Behavioral and Experimental Economics 2017; 66:137–49.

[22] Korenok O, Millner EL, Razzolini L. Taking aversion. Journal of Economic Behavior & Organization 2018; 150:397–403.

[23] Dreber A, Ellingsen T, Johannesson M, Rand DG. Do people care about social context?: Framing effects in dictator games. Exp Econ 2013; 16(3):349–71.

[24] Kettner S E, Ceccato S. Framing matters in gender-paired dictator games. Working Paper No. 557; 2014.

[25] Smith A. On the nature of pessimism in taking and giving games. Journal of Behavioral and Experimental Economics 2015; 54:50–7.

[26] Chowdhury SM, Jeon JY, Saha B. Gender Differences in the Giving and Taking Variants of the Dictator Game. Southern Economic Journal 2017; 84(2):474–83.

[27] Oxoby RJ, Spraggon J. Mine and yours: Property rights in dictator games. Journal of Economic Behavior & Organization 2008; 65(3–4):703–13.

[28] Krupka EL, Weber RA. Identifying Social Norms using Coordination Games: Why does Dictator Game sharing vary? Journal of the European Economic Association 2013; 11(3):495–524.

[29] Korenok O, Millner EL, Razzolini L. Taking, giving, and impure altruism in dictator games. Exp Econ 2014; 17(3):488–500.

[30] Brosig-Koch J, Riechmann T, Weimann J. The dynamics of behavior in modified dictator games. PLoS ONE 2017; 12(4):e0176199. doi: 10.1371/journal.pone.0176199 28448506 PMC5407812

[31] List JA. On the Interpretation of Giving in Dictator Games. Journal of Political Economy 2007; 115(3):482–93.

[32] Bardsley N. Dictator game giving: Altruism or artefact? Exp Econ 2008; 11(2):122–33.

[33] Goswami I, Urminsky O. When should the Ask be a Nudge?: The Effect of Default Amounts on Charitable Donations. Journal of Marketing Research 2016; 53(5):829–46.

[34] Fiala L, Noussair CN. Charitable Giving, Emotions, and the Default Effect. Econ Inq 2017; 55(4):1792–812.

[35] Alt M, Gallier C, Schlüter A, Nelson K, Anggraini E. Giving to versus Taking from In- and Out-Group Members. Games 2018; 9(3):57.

[36] Henrich J, Heine SJ, Norenzayan A. The weirdest people in the world? Behav Brain Sci 2010; 33(2–3):61–83; discussion 83–135. doi: 10.1017/S0140525X0999152X 20550733

[37] Henrich J. The WEIRDest People in the World: How the West Became Psychologically Peculiar and Particularly Prosperous. Farrar, Straus and Giroux 2020.

[38] Brañas-Garza P. Poverty in dictator games: Awakening solidarity. Journal of Economic Behavior & Organization 2006; 60(3):306–20.

[39] Bachke ME, Alfnes F, Wik M. Information and donations to development aid projects. Journal of Behavioral and Experimental Economics 2017; 66:23–8.

[40] Aguiar F, Brañas-Garza P, Miller LM. Moral distance in dictator games. Judgment and Decision Making 2008; 3(4):344–54.

[41] Andreoni J. Giving with Impure Altruism: Applications to Charity and Ricardian Equivalence. Journal of Political Economy 1989; 97(6):1447–1458.

[42] Konow J. Mixed feelings: Theories of and evidence on giving. Journal of Public Economics 2010; 94(3–4):279–97.

[43] DellaVigna S, List JA, Malmendier U. Testing for altruism and social pressure in charitable giving. The Quarterly Journal of Economics 2012; 127(1):1–56. doi: 10.1093/qje/qjr050 22448394

[44] Andreoni J, Bernheim BD. Social Image and the 50–50 Norm: A Theoretical and Experimental Analysis of Audience Effects. Econometrica 2009; 77(5):1607–36.

[45] Fischbacher U. z-Tree: Zurich toolbox for ready-made economic experiments. Exp Econ 2007; 10(2):171–8.

[46] Greiner B. Subject pool recruitment procedures: Organizing experiments with ORSEE. J Econ Sci Assoc 2015; 1(1):114–25.

[47] Engel C. Dictator games: A meta study. Exp Econ 2011; 14(4):583–610.

[48] Hayek FA. Law, legislation, and liberty: A new statement of the liberal principles of justice and political economy. Chicago, London: The University of Chicago Press; 2021. (The collected works of F. A. Hayek volume XIX).

[49] Thaler RH, Sunstein CR. Nudge: Improving decisions about health, wealth, and happiness. Yale University Press; 2008.

