# Supplementary material for: Charitable giving: The role of framing and information
Source: PLoS One. 2023 Jul 11;18(7):e0288400. doi: 10.1371/journal.pone.0288400 (PMC10335672; doi:10.1371/journal.pone.0288400)
Supplement: S1 Appendix — (DOCX) [file pone.0288400.s001.docx]

**Appendix A**

## **Charitable giving in a traditional theoretical utility-maximization model**

Consider a slightly modified theoretical utility-maximization framework originally as provided in [42]. It allows for both motivation by (impersonal) social norms of giving and a (personal) warm glow of giving [41]. The utility U of the donor who adopts the dictator role is given by

|  | $U=u\left( e-x \right)- f(x-\varphi) + \alpha g(x)$ | (1) |
| --- | --- | --- |

where, $e$ is the dictator’s endowment, and *x* is the amount that the dictator donates to the charity. For$u(e-x),$ which is the utility the dictator derives from material payoff to herself, it is assumed: $u’(.) > 0$, $u’’(.) < 0$. The second term $f(.)$ represents the disutility the dictator suffers from deviating with her donation from the contextually prevailing giving norm, $\varphi$; assuming that $-f\left( . \right)<0$ is strictly concave in $x$ and adopting its maximum (i.e., its minimum in absolute terms) when the dictator fully complies with the norm (x = $\varphi)$. The assumption here is that norm compliance is associated with positive feelings and violation of norms with negative feelings. The third term relates to the ‘warm glow’ of giving [41, 43], with $g’(.) > 0$ and $g’’(.) < 0$ (for simplicity assuming that the warm glow of not taking is identical to the warm glow of giving). The parameter, $\alpha\geq0$, represents the intensity of the warm glow of giving. Following [38], the parameter $\alpha$ can be increased by moral-distance-reducing information about the charity.

In a standard dyadic dictator game, the typical assumption is a norm of giving 50 percent of the endowment if the recipient has no own endowment [44]. If the recipient is a charity, the norm might be higher. However, as long as it is below 100 percent, whatever the norm of giving in a giving framework, we assume that the norm is higher in a taking framework. This is supported by an observation of [28] who find that an allocation that leaves the recipient (in their case another person) with less than half of the endowment is perceived as less socially appropriate under the taking frame than under the giving frame.

In view of the preceding it becomes straightforward to demonstrate theoretically (see, [43], Proposition 4 and the Appendix) that a utility-maximizing dictator’s donation changes in direct relationship to – though by less than – a change in the giving norm:

|  | $0 <$ $\frac{dx^{*}}{d\varphi}$ $<1$ | (2) |
| --- | --- | --- |

where $x^{*}$ is the solution to the dictator’s utility maximization task. Since

|  | $\frac{dx^{*}}{d\varphi}= \frac{-f’’}{u’’- f’’+\alpha g’’}$ | (3) |
| --- | --- | --- |

it is also straightforward that increasing the warm-glow factor $\alpha$ from $\alpha^{L}$ to $\alpha^{H}$, reduces the effect of the norm change:

|  | $\frac{-f’’}{u’’- f’’+ \alpha^{H}g’’}<\frac{-f’’}{u’’- f’’+ \alpha^{L}g’’}$ | (4) |
| --- | --- | --- |

Applying the implicit function theorem to solve for$x = x^{*}(\alpha)$, substituting this into the first-order condition for utility maximization and differentiating with respect to $\alpha$, we get for the effect of the warm-glow factor:

|  | $\frac{dx^{*}}{d\alpha}= \frac{g'}{f^{''}-u^{''}- \alpha g''} >0$ | (5) |
| --- | --- | --- |

From this we can derive the following hypotheses.

H1: Ceteris paribus, donations will be higher under the taking frame than under the giving frame.

This is due to our assumption that moving from a giving to a taking frame increases the norm $\varphi$ and relation (2) implying that the optimal donation $x^{*}$increases in $\varphi$.

H2: Ceteris paribus, donations will be higher, the more (positive) information about the charity is provided.

This is due to our assumption that information about the charity increases the warm-glow factor $\alpha$ and equation (5) implying that the optimal donation $x^{*}$increases in $\alpha$.

H3: The more detailed the information about the charity, the smaller will be the impact of the taking frame compared with the giving frame on donations.

This directly follows from relation (4).

**Appendix B**

**Instructions**

General instructions to be read aloud in the waiting room (English/German):

| **English** | **German** |
| --- | --- |
| You are participating in an economic experiment on decision-making. You can earn money dependent on your decisions. You make your decisions anonymously and isolated from the other participants. From now on, we ask you not to speak to any other participant until the experiment is over. Please switch off your mobile phones and put them away. | Sie nehmen an einem wirtschaftswissenschaftlichen Entscheidungsexperiment teil. Abhängig von Ihren Entscheidungen können Sie bares Geld verdienen. Sie treffen dazu Ihre Entscheidungen anonym und isoliert von anderen. Ab jetzt, bitten wir Sie nicht mehr mit anderen zu kommunizieren, bis das Experiment beendet ist. Bitte schalten Sie zudem Ihre Mobiltelefone aus und stecken Sie sie weg. |
| In the course of the experiment, you will be asked to complete a survey. Please complete the survey as thoroughly as possible. The survey will be displayed to you in a computer in the neighboring room. If you have a question while completing the survey, please come forward individually. | Im Laufe des Experiments werden Sie gebeten einen Fragebogen auszufüllen. Füllen Sie den Fragebogen bitte so gewissenhaft wie möglich aus. Der Fragebogen wird Ihnen an einem Computer im Nachbarraum angezeigt. Falls Sie während des Ausfüllens eine Frage haben, so kommen Sie bitte einzeln nach vorne. |
| After the experiment, money might be transferred to the *International Federation of the Red Cross and Red Crescent* (IFRC). The *German Red Cross* among others belongs to this organization. | Nach dem Experiment wird gegebenenfalls Geld an die *Internationale Rotkreuz- und Rothalbmond-Bewegung* (IFRC) transferiert. Zu dieser Organisation gehört unter anderem das *Deutsche Rote Kreuz*. |

| **<*some* information and *much* information environment only:>**  [The *International Federation of the Red Cross and Red Crescent* (IFRC) is the world's largest humanitarian network. Their website is accessible via www.ifrc.org. Together with its 190 national societies they are focusing their work in three key areas: 1) disaster response and recovery, 2) development and 3) promoting social inclusion and peace. Their task is to coordinate in the case of an international catastrophe, the promotion of the cooperation between the national societies and the representation of the national societies in the international context.] | [Die *Internationale Rotkreuz- und Rothalbmond-Bewegung* ist das weltweit größte humanitäre Netzwerk. Ihre Internetpräsenz ist unter www.ifrc.org erreichbar. Zusammen mit ihren 190 nationalen Gesellschaften fokussiert sie sich auf drei Kernbereiche: 1.) Hilfe und Wiederaufbau bei Katastrophen. 2.) Entwicklungsarbeit und 3.) Förderung von Frieden und sozialer Inklusion. Ihre Aufgabe ist die Koordination im internationalen Katastrophenfall, die Förderung der Kooperation zwischen den nationalen Gesellschaften und die Repräsentation der nationalen Gesellschaften im internationalen Kontext.] |
| --- | --- |
| We are not connected to the organization. However, you can be sure that the transferred money actually reaches the organization.  A receipt will be posted on the bulletin board of the chair of microeconomics after the conclusion of the experiment. | Wir stehen in keiner Verbindung zu dieser Organisation. Sie können sich jedoch sicher sein, dass das transferierte Geld die Organisation tatsächlich erreicht. Eine Quittung wird nach Abschluss des Experiments am Schwarzen Brett der Professur für Mikroökonomik ausgehängt. |
| **<*much* information environment only:>**  [We are now showing you a video, that points out the fundamental principles of the *International Federation of the Red Cross and Red Crescent.* The video is freely available on www.youtube.com.] | [Wir zeigen Ihnen nun ein Video, das die Grundsätze der *Internationale Rotkreuz- und Rothalbmond-Bewegung* aufzeigt. Das Video ist auf www.youtube.com frei verfügbar.] |
| We now ask you to go to the computer with your participant number. Please close the curtain and keep it close until the end of the experiment. This ensures that you are not observed during the survey. To start the survey, you must click the Next button. Thank you for your participation! | Wir bitten Sie nun, sich zu dem Computer mit Ihrer Teilnehmernummer zu begeben. Bitte schließen Sie den Vorhang und halten Sie ihn bis zum Ende des Experiments geschlossen. Dies gewährleistet, dass Sie während Ihren Entscheidungen unbeobachtet sind. Um mit dem Fragebogen zu beginnen, müssen Sie auf die <Weiter>-Taste klicken. Vielen Dank für Ihre Teilnahme! |

Transcript of the video (English/German):

| **English** | **German** |
| --- | --- |
| Humanity  The International Red Cross and Red Crescent Movement, born of a desire to bring assistance without discrimination to the wounded on the battlefield, endeavors, in its international and national capacity, to prevent and alleviate human suffering wherever it may be found. Its purpose is to protect human life and health and to ensure respect for the human being. It promotes mutual understanding, friendship, cooperation and lasting peace amongst all people. | Menschlichkeit  Die internationale Rotkreuz- und Rothalbmond-Bewegung, entstanden aus dem Willen, den Verwundeten der Schlachtfelder unterschiedslos Hilfe zu leisten, bemüht sich in ihrer internationalen und nationalen Tätigkeit, menschliches Leiden überall und jederzeit zu verhüten und zu lindern. Sie ist bestrebt, Leben und Gesundheit zu schützen und der Würde des Menschen Achtung zu verschaffen. Sie fördert gegenseitiges Verständnis, Freundschaft, Zusammenarbeit und einen dauerhaften Frieden unter allen Völkern. |
| Impartiality  It makes no discrimination as to nationality, race, religious beliefs, class or political opinions. It endeavors to relieve the suffering of individuals, being guided solely by their needs, and to give priority to the most urgent cases of distress. | Unparteilichkeit  Die Rotkreuz- und Rothalbmond-Bewegung unterscheidet nicht nach Nationalität, Rasse, Religion, sozialer Stellung oder politischer Überzeugung. Sie ist einzig bemüht, den Menschen nach dem Maß ihrer Not zu helfen und dabei den dringendsten Fällen den Vorrang zu geben. |
| Neutrality  In order to continue to enjoy the confidence of all, the movement may not take sides in hostilities or engage at any time in controversies of a political, racial, religious or ideological nature. | Neutralität  Um sich das Vertrauen aller zu bewahren, enthält sich die Rotkreuz- und Rothalbmond-Bewegung der Teilnahme an Feindseligkeiten wie auch, zu jeder Zeit, an politischen, rassischen, religiösen oder ideologischen Auseinandersetzungen. |

| Independence  The Movement is independent. The National Societies, while auxiliaries in the humanitarian services of their governments and subject to the laws of their respective countries, must always maintain their autonomy so that they may be able at all times to act in accordance with the principles of the Movement. | Unabhängigkeit  Die Rotkreuz- und Rothalbmond-Bewegung ist unabhängig. Wenn auch die Nationalen Gesellschaften den Behörden bei ihrer humanitären Tätigkeit als Hilfsgesellschaften zur Seite stehen und den jeweiligen Landesgesetzen unterworfen sind, müssen sie dennoch eine Eigenständigkeit bewahren, die ihnen gestattet, jederzeit nach den Grundsätzen der Rotkreuz- und Rothalbmond-Bewegung zu handeln. |
| --- | --- |
| Voluntary service  It is a voluntary relief movement not prompted in any manner by desire for gain. | Freiwilligkeit  Die Rotkreuz- und Rothalbmond-Bewegung verkörpert freiwillige und uneigennützige Hilfe ohne jedes Gewinnstreben. |
| Unity  There can be only one Red Cross or one Red Crescent Society in any one country. It must be open to all. It must carry its humanitarian work throughout its territory. | Einheit  In jedem Land kann es nur eine einzige Nationale Rotkreuz- oder Rothalbmond-Gesellschaft geben. Sie muss allen offen stehen und ihre humanitäre Tätigkeit im ganzen Gebiet ausüben. |
| Universality  The International Red Cross and Red Crescent Movement. In which all Societies have equal status and share equal responsibilities and duties in helping each other, is worldwide. | Universalität  Die Rotkreuz- und Rothalbmond-Bewegung ist weltumfassend. In ihr haben alle Nationalen Gesellschaften gleiche Rechte und die Pflicht, einander zu helfen. |

On screen instructions (English/German):

| **English** | **German** |
| --- | --- |
| You will find 10 euros on the mat. These are destined for you [for the IFRC]. On this mat there are three 2 euro coins, two 1 euro coins, five 20 cents coins and ten 10 cent coins. Please count the money and put it back on the mat. | Auf der Matte vor Ihnen finden Sie 10 Euro. Diese sind für Sie [für die Internationale Rotkreuz- und Rothalbmond-Bewegung] bestimmt. Auf dieser Matte befinden sich drei 2 Euro Münzen, zwei 1 Euro Münzen, fünf 20 Cent Münzen und zehn 10 Cent Münzen. Bitte zählen Sie das Geld nach und legen es anschließend zurück auf die Matte. |
| While filling in the survey, you will have once the opportunity to reduce your initial endowment in order to increase the amount dedicated to the IFRC. [… to reduce the IFRC’s initial endowment in order to increase the amount dedicated to you.] No other participant will know how you decided. Regardless of how you decide, you will have to wait 30 seconds to complete the survey. | Während des Ausfüllens des Fragebogens werden Sie 1x die Möglichkeit erhalten, den für Sie vorgesehenen Betrag auf der Matte zu reduzieren, um damit den Betrag für die Internationale Rotkreuz- und Rothalbmond-Bewegung zu erhöhen. [… den für die Internationale Rotkreuz- und Rothalbmond-Bewegung vorgesehenen Betrag auf der Matte zu reduzieren, um damit den Betrag für Sie zu erhöhen.] Kein anderer Teilnehmer wird erfahren, wie Sie sich entschieden haben. Unabhängig davon, wie Sie sich entscheiden, müssen Sie 30 Sekunden warten, bis Sie den Fragebogen weiter ausfüllen können. |

Decision stage (English/German):

| **English** | **German** |
| --- | --- |
| You have now the opportunity to reduce your endowment in order to increase the amount dedicated to the IFRC. [… to reduce the endowment of the IFRC in order to increase the amount dedicated to you.]  Before your decision:  The amount dedicated to you in EUR: 10.00 [0.00]  The amount dedicated to the organization in EUR: 0.00 [10.00]  Please enter how much you would like to transfer from your initial endowment to the account of the organization. [… from the charity’s initial endowment to your account.] Enter an amount between 0 euros and 10.00. Choose an amount rounded to 0.10 euro.  Transferred amount (in EUR): ____  After the 30 seconds, you can confirm the amount by clicking OK. | Sie haben nun die Möglichkeit den für Sie bestimmten Betrag zu reduzieren, um damit den Betrag für die Internationale Rotkreuz- und Rothalbmond-Bewegung zu erhöhen. [… den für die Internationale Rotkreuz- und Rothalbmond-Bewegung bestimmten Betrag zu reduzieren, um damit den Betrag für Sie zu erhöhen.]  Vor Ihrer Entscheidung:  Der für Sie bestimmte Betrag in Euro: 10.00 [0.00]  Der für die Organisation bestimmte Betrag in Euro: 0.00 [10.00]  Bitte tragen Sie ein, wie viel Sie von dem für Sie bestimmten Betrag der Organisation übertragen möchten. [… wie viel Sie von dem für die Organisation bestimmten Betrag Ihnen selbst übertragen möchten.] Tragen Sie dazu einen Betrag zwischen 0 Euro und 10.00 Euro ein. Wählen Sie einen auf 0,10 Euro gerundeten Betrag.  Übertragener Betrag (in Euro): ____  Nach Ablauf der 30 Sekunden können Sie den Betrag mit einem Klick auf OK bestätigen. |

**Photos (money on the mat)**


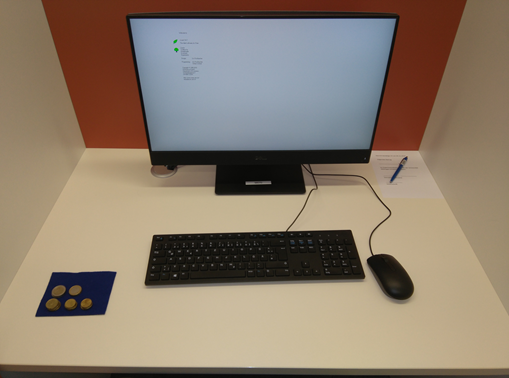


Cubicle with computer, keyboard, mouse, money, a pen and the receipt.


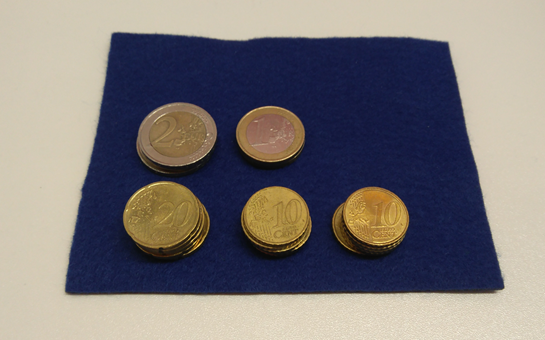


Presentation of the money.
